# Face masks have a limited effect on the feeling of being looked at

**DOI:** 10.3389/fnins.2022.1028915

**Published:** 2022-11-29

**Authors:** Janek S. Lobmaier, Daria Knoch

**Affiliations:** Department of Social Neuroscience and Social Psychology, Institute of Psychology, University of Bern, Bern, Switzerland

**Keywords:** cone of direct gaze, CoDG, hygienic face mask, eye gaze, mutual gaze

## Abstract

**Introduction:**

Wearing face masks has been promoted as an effective measure to reduce the spread of COVID-19. Because face masks cover a major part of the face, they have detrimental effects on various aspects of social cognition. Yet, a highly important feature of the face is not occluded by face masks: the eyes. The eyes play an important role in social interactions: knowing where another person is looking is of central importance when interacting with others. Recent research has reported an attentional shift toward the eye region as a consequence of the widespread exposure to face masks. However, no study has yet investigated the influence of face masks on the perception of eye gaze direction. Here we investigated whether face masks have an effect on the feeling of being looked at. Assuming an attentional shift toward the eyes, we might expect more accurate gaze perception in faces wearing face masks.

**Methods:**

Sixty-five participants decided for a series of realistic avatar faces whether each face was making eye contact or not. Half of the faces wore face masks, the other half did not. For each participant and separately for each condition (mask vs. no mask), we calculated the cone of direct gaze (CoDG), a commonly used measure to quantify the range of gaze angles within which an observer assumes mutual gaze.

**Results:**

Contrary to our expectations, results show that mutual gaze is not recognized more accurately in masked faces. Rather, the CoDG was, on average, slightly wider for faces wearing masks compared to faces without masks.

**Discussion:**

Notwithstanding the relatively small effect of face mask, these findings potentially have implications on our social interactions. If we inadvertently feel looked at by an onlooker, we may react inappropriately by reciprocating the alleged approach orientation.

## Introduction

Knowing where another person is looking is of central importance for social interactions (Argyle and Cook, 1976; Kleinke, 1986; Baron-Cohen, 1995) since the direction of eye gaze portrays information about other people’s focus of attention. Of special importance is the skill to distinguish between mutual and averted eye gaze. When someone looks us in the eye, we may be invited to reciprocate the affiliative orientation, which increases the chance of a social interaction.

The eyes are the most salient and perhaps the biologically most relevant features of a face. Already newborn infants show a preference for the eye region (Farroni et al., 2002) and already at the age of 4 months babies can discriminate between direct and averted gaze (Farroni et al., 2004). It is therefore unsurprising that humans are good at distinguishing between gaze that is averted and mutual gaze (e.g., Gibson and Pick, 1963; Cline, 1967; Gale and Monk, 2000). Despite this generally accurate ability to detect eye contact, various research has demonstrated a considerable range of gaze directions which are perceived as being direct (Gamer and Hecht, 2007; Harbort et al., 2017; Balsdon and Clifford, 2018). Gamer and Hecht (2007) hence suggested using the metaphor of a cone to describe the perception of gaze direction, rather than that of a ray as assumed in earlier studies (e.g., Gale and Monk, 2000; Symons et al., 2004).

The cone of direct gaze (CoDG) describes the range of gaze angles which an observer perceives as making eye contact. Previous research has shown that most people have a rather wide CoDG, meaning they interpret a rather large range of gaze angles as being direct. By accepting a relatively large range of gaze directions to be making eye contact, observers avoid the cost of missing direct gaze, which is greater that mistakenly interpreting averted gaze as direct (Langton et al., 2004).

Since the outbreak COVID-19 pandemic, governments and health authorities around the globe recommend wearing hygienic face masks as an effective measure to reduce the spread of the disease. Because hygienic face masks cover a major part of the face, they substantially impair face perception (Freud et al., 2020; Marini et al., 2021; Noyes et al., 2021) and emotion recognition (Carbon, 2020; Grundmann et al., 2021; Marini et al., 2021; Noyes et al., 2021; Grahlow et al., 2022). Similarly, Fitousi et al. (2021) found that masks hindered the perception of face identity, emotional expression, age and gender of a face, both in terms of accuracy and speed. The difficulty to correctly “read” faces as a result of face covering leads to detrimental effects on various aspects of social cognition, such as establishing and maintaining effective interpersonal social interactions (Mheidly et al., 2020; Spitzer, 2020). Specifically, wearing face masks affects inter-personal distance regulation (e.g., Cartaud et al., 2020; Kroczek et al., 2022) and the perceived trustworthiness of others (e.g., Oldmeadow and Koch, 2021). However, it seems that people have accustomed to the fact that half the face is covered by a mask. Mheidly et al. (2020) assume that as a consequence of the wide use of face masks in recent years, the visible eye region becomes more important. This was confirmed by recent work of Barrick et al. (2021), who demonstrated that people with higher levels of mask exposure make more use of cues from the eye region when processing emotional facial expressions than people with less mask exposure. Further, as exposure to masks increased, those with the most social interactions also experienced the greatest increase in the use of information from the eye region (Barrick et al., 2021). These results provide evidence that the perception of facial cues shows a certain plasticity. As a consequence of the widespread exposure to face masks, an attentional shift has occurred in how people process faces: they have learnt to direct their attention more to the eye area of the face. It seems evident that during the COVID-19 epidemic a change has occurred in the way we perceive and interpret faces, through the interaction with mask-wearing counterparts.

Given that the recommendation to wear face masks during the COVID-19 pandemic poses challenges on our non-verbal communication, we investigated whether face masks have an effect on the perception of mutual gaze. We use the term “mutual gaze” to describe the situation in which somebody is making eye contact with an observer without distinguishing between gaze and head direction. Following this definition, gaze direction was always aligned with the head orientation in the present study and the head as a whole was rotated (cf. Gianotti et al., 2018; Lobmaier et al., 2021). We measured the CoDG to quantify the range of gaze angles within which an observer assumes mutual gaze (cf. Gamer and Hecht, 2007; Ewbank et al., 2009; Gamer et al., 2011; Harbort et al., 2017; Gianotti et al., 2018). Because people will resort to information contained in the visible eye region when the lower part of the face is covered, we expect the CoDG to be narrower (i.e., perception of mutual gaze to be more accurate) in faces wearing hygienic masks. By covering other prominent facial parts (e.g., mouth, cheeks, and nose), face masks may result in less distraction from the eyes or might generally make the eyes more salient, again leading to more accurate gaze perception. Because the alleged attentional shift toward the eye region seems to depend on the amount of exposure to face masks in everyday life (cf. Barrick et al., 2021), we also assessed and controlled for the amount of exposure to face masks using the questionnaire introduced by Barrick et al. (2021).

## Materials and methods

### Participants

Sixty-five participants (17 men, 48 women) aged between 19 and 29 years (*M* = 22.9, SD = 2.4) volunteered to take part in this study for course credit or a snack. All participants had normal or corrected to normal vision. Sixty participants were of Caucasian decent, five were of other ethnicities (Asian, African, or South-American). All participants reported that they lived and were brought up in Central Europe. In three cases the CoDG could not be estimated due to inconsistent responses recorded during the task (two in the “mask” condition and one in the “no mask” condition). The study was approved by the local Ethics Committee. All participants gave written informed consent and were informed of their right to discontinue participation at any time. Data were collected in a single wave and then analyzed (no analyses were calculated before all participants were tested).

### Stimuli

Three-dimensional face stimuli were created using the software package FaceGen Modeller 3.5.2 (Singular Inversions Inc., 2010) which enables the generation of face stimuli with a high level of realism. Faces of four Caucasian gender-neutral avatars showing a neutral expression were generated (FOV Angle = 17; Distance Ratio = 3). To ensure that the perceptual features of different face stimuli did not affect the results, the four avatars were generated by using the “genetic” tool. This tool allows to create highly similar faces with a predefined level of randomness (30%). The gaze direction of the faces was aligned with the head direction, so that nose and gaze fixation point lay on the same axis. The avatar heads obtained with this procedure were then rotated in 1° steps producing 17 different viewing angles (from 1° to 8° to the left and right, and 0°). For the “facemask” condition, a surgical face mask was superimposed on each avatar face using Adobe Photoshop 2021.

### Task and procedure

**FIGURE 1:**
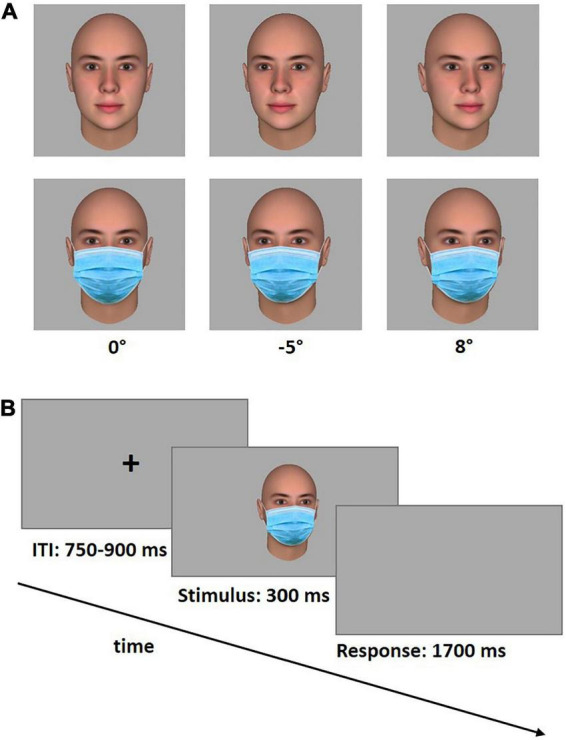
Stimulus examples in three different viewing angles (0°, –5°, and 8°), with and without face mask **(A)** and schematic time line of the gaze task **(B)**. The task consisted of a variable inter-stimulus interval (ITI), followed by a stimulus face (300 ms), which was then replaced with a response window (1,700 ms). Participants responded whether or not the stimulus face was looking at them *via* two orthogonally arranged custom-made response buttons.

After obtaining written informed consent, participants were seated comfortably in a dimly lit room and received written instructions for the gaze discrimination task. They sat a distance of approximately 60 cm from a PC screen. The face stimuli appeared on the screen with a width of 6 cm, thus subtending a visual angle of approximately 5.7°. This corresponds to a distance of approximately 180 cm in real life. Lighting conditions were kept constant for all participants and the screen position was manually adapted so that the eyes of the avatars were vertically aligned with the eyes of the participants. We used an established gaze perception paradigm (cf. Gianotti et al., 2018; Lobmaier et al., 2021) where each participant saw a series of avatar faces and decided for each face whether it was making eye contact or not. Half of the avatar faces wore face masks, the other half did not. Each trial started with the presentation of a fixation cross for a variable duration (between 750 and 900 ms) followed by a stimulus face (300 ms). After this, participants had 1,700 ms to answer. Participants were asked to decide as quickly as possible whether the presented face was gazing directly at them using predefined buttons on a custom made response box. A schematic timeline of the gaze discrimination task is shown in Figure 1. The keys on the response box were aligned perpendicular to each other to avoid any gaze induced response biases. The correspondence between yes/no keys and which hand was used for yes/no was counterbalanced across participants. The gaze discrimination task comprised 288 trials [18 angles (0° angle was shown twice) × 4 avatars × 2 repetitions × 2 experimental conditions (mask vs. no mask)]. Masked and unmasked stimuli were presented blockwise in an ABABAB/BABABA fashion where A is the “no mask” condition and B is the “facemask” condition. Half of the participants started with the “no mask” condition, the other half with the “facemask” condition. Each block contained 48 trials which were presented pseudorandomly within each block, with the constraint that each angle and face identity was equally distributed across the blocks.

After the gaze task which took approximately 15–20 min to complete, participants filled in the questionnaire introduced by Barrick et al. (2021) assessing the amount of exposure to face masks during the pandemic.

### Statistical analyses

#### Cone of direct gaze calculation

The proportion of yes and no responses across visual angles were used to compute the CoDG. In a first step we calculated the percentage of times the participant decided that the face stimulus was looking directly at him/her as a function of the gaze angle, separately for each mask condition. Using R statistics software (R Core Team, 2021), we then fitted the data to a logistic function to calculate the points of subjective equivalence (PSE). PSE is defined as the angle at which a participant would be predicted to assume eye contact or no eye contact with equal frequency (i.e., 50%). We calculated the PSE separately for faces rotated to the left and right. The CoDG was calculated as the sum of the absolute values of the left and right side PSE.

#### Testing the effect of face masks on the cone of direct gaze

Any outliers were winsorized before further analyses (Dixon, 1960). Specifically, outliers more than three standard deviations from the mean were substituted with the highest observed value that was within three standard deviations. This was the case for one data point in the mask condition and one in the no-mask condition.

Linear mixed models (LMM) were run using the R package lme4 (Bates et al., 2015), while the package lmerTest (Kuznetsova et al., 2017) was used to determine the significance of the predictors. LMMs are advantageous over ANOVAs when the data-set is unbalanced or when there are missing values. After calculating the intraclass correlation, to check the adequacy of an LMM, a random-intercept model was estimated. In this model, only the level 1 predictor “condition” was included in the model as a fixed effect, with CoDG as the dependent variable and a random intercept for participants. As suggested by LaHuis et al. (2014), the explained variance (R2) by condition was calculated using this random intercept model. In a second step, we additionally entered the predictors participant sex and amount of exposure (full model).

## Results

**FIGURE 2:**
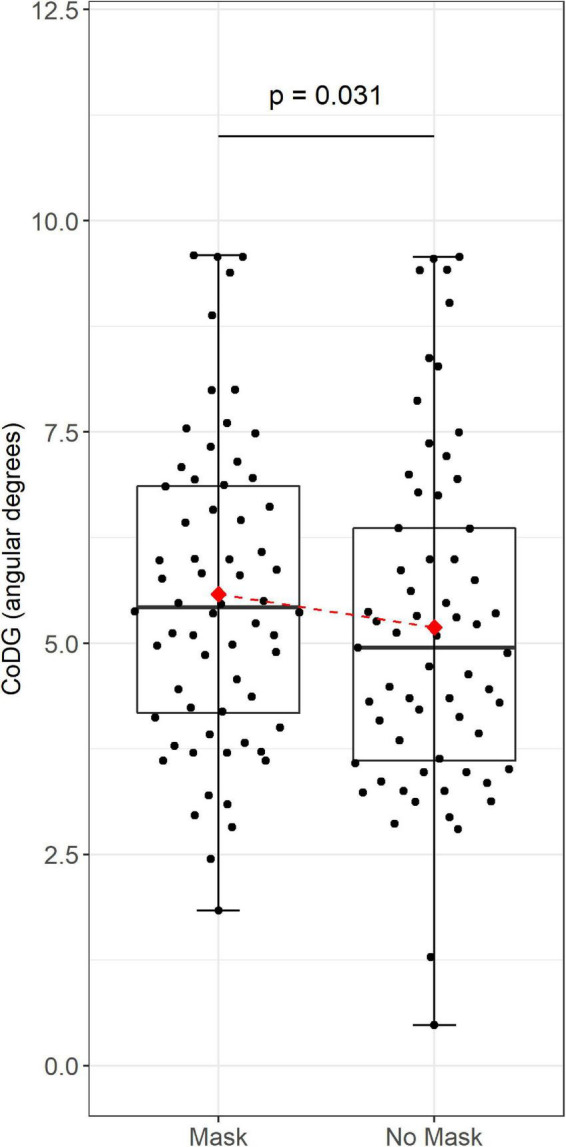
Data are plotted as box plots for each condition (“mask” and “no mask”). Bold horizontal lines indicate median values, boxes indicate 25/75% interquartile range, and whiskers indicate 1.5 × interquartile range. Red diamonds indicate the mean cone of direct gaze (CoDG) in the “mask” and “no mask” condition. Red line connects mean CoDG in “mask” and “no mask” condition. Individual CoDG are shown separately as black dots, jittered proportionally to the density (jittered density plot).

In the mask condition, the CoDG ranged from 1.84° to 9.59° (mean = 5.58°), in the no-mask condition, the CoDG ranged from 0.48° to 9.57° (mean = 5.19°) (see Figure 2). The intraclass correlation of 0.86 revealed substantial differences in CoDG between participants. Regarding the fixed effect, the results of the LMM with CoDG as the dependent variable and the mask condition as the predictor revealed a main effect of mask condition (estimate = −0.280; standard error = 0.126; 95% CI [−0.53, −0.03]; *t* = −2.229, df = 60.89; *p* = −0.03). Therefore, on average, the CoDG for faces without masks were 0.28 degrees narrower than for faces with a mask. The predictor condition explained approximately 5.2% of the level-1 variance within individuals of CoDG. The full model with CoDG as the dependent variable and mask condition, participant sex and exposure as predictors, and participant as random factor again revealed a significant effect of mask condition (*p* = −0.030), but no effect of participant sex (*p* = −0.821) and no effect of amount of exposure to face masks (*p* = −0.179; see Table 1). This full model explained 5.4% of the variance, only slightly more than condition alone.

**TABLE 1:** Fixed effects parameter estimates.

			95% Confidence interval				
Effect	Estimate	SE	Lower	Upper	df	*t*	*p*
(Intercept)	4.40	0.90	4.95	5.86	62.50	4.87	<0.001
Condition (No mask–mask)	–0.28	0.13	–0.53	–0.03	61.03	–2.22	**0.030**
Part sex (male–female)	–0.12	0.53	–1.29	1.25	62.29	–0.28	0.821
Exposure to face masks	0.00	0.00	0.00	0.01	61.76	1.36	0.179
Condition × exposure	0.00	0.00	0.00	0.00	60.10	–0.34	0.734

Estimate, unstandardised regression coefficients; SE, standard error; df, degree of freedom. Significant *p*-value for Condition in bold type.

## Discussion

The use of hygienic face masks pose challenges on our social interactions because they cover a major part of the face. In the present study, we investigated whether face masks have an effect on the interpretation of mutual eye gaze. Because facemasks cover the lower part of the face but spare information contained in the eye region, we assumed that, when looking at faces wearing hygienic masks, people would resort more strongly to the eyes than when the whole face is visible, leading to more accurate gaze perception. Contrary to our expectations, this was not the case: our results indicate a small but significant widening of the CoDG for faces with masks compared to faces without masks. On average, in the mask condition, people more often assumed mutual gaze when the faces were actually averted than in the no-mask condition. Closer inspection of the data revealed that this was the case for 61% of participants, whereas for 39% of the participants the CoDG got narrower in the mask condition. So, even though the mask effect is statistically significant, it did not occur for all participants and the overall effect is relatively small: the mask condition explained approximately 5.2% of the variance only. Moreover, the amount of exposure to face masks in everyday life did not influence the CoDG, neither in the mask nor in the no-mask condition, suggesting that people with higher levels of mask exposure do not make more use of cues from the eye region when processing eye gaze than people with less mask exposure.

Our findings suggest that the alleged attention shift toward the eye region as a result of high exposure to face masks had only limited effect on the CoDG. This finding is somewhat in line with a recent study by Dalmaso et al. (2021), who explored the potential impact of face masks on gaze induced attentional shifts. Using a gaze cueing paradigm in which the centrally presented cue either was a face wearing a hygienic face mask or a face without a mask, they found that face masks had no impact on cueing of attention. But even if attention were drawn to the eyes, a predominant uncertainty remains when encountering a person wearing a mask. Wearing masks increases the ambiguity of social interactions and this naturally leads to even more uncertainty. This increased uncertainty may also make it more difficult to interpret gaze direction. As a result, people may tend to interpret ambiguous gaze lines as making eye contact. Indeed, previous research indicates that as ambiguity increases, gaze is more likely to be judged as directed toward oneself (Mareschal et al., 2013; Balsdon and Clifford, 2018). A different study found that stressed people tend to interpret a wider range of gaze lines as making eye contact than unstressed people (Rimmele and Lobmaier, 2012). This interpretation is consistent with the evolutionary informed view that in cases of ambiguity or heightened stress, it is safer to assume eye contact when the looker is actually averting her gaze than to mistakenly interpret direct gaze as being averted (Langton et al., 2004).

Wearing face masks seems to induce an additional bias on the perception of mutual gaze (at least in most people), making it more likely to experience mutual gaze. This may be because the mask disguises other important cues to gaze direction, such as the direction of the nose and mouth (cf. Langton et al., 2004). Alternatively, the widened CoDG for faces wearing hygienic masks could be due to the fact that social interactions with mask-wearing individuals are generally more challenging due to impaired face recognition (Freud et al., 2020; Marini et al., 2021; Noyes et al., 2021) and impaired emotion recognition (Carbon, 2020; Grundmann et al., 2021; Marini et al., 2021; Noyes et al., 2021; Grahlow et al., 2022).

Finally, a further possible explanation for wider CoDG in masked faces is that hygienic masks alter perceived attractiveness of a face. Indeed, recent research found that faces wearing face masks are perceived as being more attractive than uncovered faces (Patel et al., 2020; Hies and Lewis, 2022). Meanwhile, gaze lines of more attractive people are more often interpreted as making eye contact than gaze lines of less attractive people, presumably due to a self-referential positivity bias (Kloth et al., 2011). So, if hygienic masks increase the attractiveness of a face and if the CoDG is wider when interpreting the gaze of an attractive compared to a less attractive face, it stands to reason that the CoDG should be wider when interacting with a person wearing a face mask. To specifically test whether the avatar faces used in the present study appear to be more attractive when wearing face masks, we conducted a follow-up study, in which 64 additional participants rated the attractiveness of each of the four avatar faces once with and once without a face mask. Faces were presented one after the other in a random order and participants used a slider to rate the attractiveness of each face on a scale ranging from 0 (very unattractive) to 100 (very attractive). Results unequivocally showed that masked faces were perceived as being more attractive (*M* = 53.5) than faces without masks (45.9), *t* = 3.83, *p* < 0.001, thus replicating previous findings (Patel et al., 2020; Hies and Lewis, 2022). More importantly, assuming that more attractive faces are more likely interpreted as making eye contact than less attractive faces (Kloth et al., 2011) the findings of the follow-up study can be taken as an indirect explanation for the slightly wider CoDG in the mask condition compared to the no-mask condition.

We note that our claims have to be treated with some caution. As mentioned above, even though the CoDG was significantly wider for faces wearing hygienic masks, the presence of face masks explained only a small proportion of the variance. This indicates that further factors contribute to the variability in the width of the CoDG. It will have to be the aim of future studies to identify these. A second noteworthy limitation is that in this study we use the term “mutual gaze” to describe the situation in which somebody is making eye contact with an observer without distinguishing between gaze and head direction (cf., Gianotti et al., 2018; Lobmaier et al., 2021). Although people certainly sometimes avert their gaze while orienting their head straight ahead, we followed the assumption that people will most likely also turn their heads toward the person they are communicating with. Thus, we varied gaze direction together with the head direction to create what we believed to be an ecologically valid situation. With this definition it is difficult to know whether the observed results are relative to a variation in the gaze direction alone or to a variation in the direction of the whole head. Because gaze direction and head orientation were aligned, facial masks may have occluded convergent information regarding the exact gaze direction, particularly the orientation of the nose. Also, our assumption that eye direction and head orientation largely coincide does not accommodate the fact that slight deviations of gaze and head direction are likely to occur in real life. Indeed, smaller corrections of the gaze direction take place without head movements. Whether and how such misalignments of gaze and head direction can influence the CoDG in masked faces is a highly interesting question that deserves further investigation. The idea that increased attention to the eye region improves accuracy of gaze perception might come into play especially when eyes and head direction are not aligned, because only then the complex interactions between head orientation and gaze direction on perceived gaze direction arise (Hecht et al., 2020, 2021). Future studies may wish to specifically disentangle the relative influence of head and eye direction.

The use of hygienic face masks is expected to continue to be part of normality even after the COVID-19 pandemic (Rab et al., 2020; Molnar-Szakacs et al., 2021), hence evidence-based research is needed that investigates the impact of mask wearing on our social interactions. While numerous studies have investigated how mask wearing affects perception of emotional facial expressions or face recognition, we are aware of no study that studied interpretation of eye gaze direction in faces wearing face masks. The present study contributes to reducing this research gap by examining whether wearing a face mask affects the feeling of being looked at. Using an established paradigm to measure the CoDG (cf., Gianotti et al., 2018; Lobmaier et al., 2021), we found that, on average, the CoDG was slightly wider when judging the gaze direction of faces with hygienic masks. This means that, at least for the majority of people, face masks can lead to biased perception of mutual eye gaze in a way that we more likely feel looked at by mask-wearers than by people without face masks. As noted above, the mask effects were rather small, suggesting that overall, the neural mechanisms responsible for detecting mutual gaze are surprisingly robust and are only minimally disrupted when half of the face is covered by a hygienic mask. Nevertheless, in our everyday lives, face masks can have an impact on our social interactions in a way that, if we inadvertently feel looked at and addressed by an onlooker, we may react inappropriately by reciprocating the alleged approach orientation.

## Data availability statement

The original contributions presented in this study are included in the article/supplementary material, further inquiries can be directed to the corresponding authors.

## Ethics statement

The studies involving human participants were reviewed and approved by the Ethics Committe of the Faculty of Human Sciences, University of Bern. The patients/participants provided their written informed consent to participate in this study.

## Author contributions

JL performed the statistical analysis and wrote the first draft of the manuscript. JL and DK contributed to conception, design of the study, manuscript revision, read, and approved the submitted version.
